# Tribological Performance Investigation of a Commercial Engine Oil Incorporating Reduced Graphene Oxide as Additive

**DOI:** 10.3390/nano11020386

**Published:** 2021-02-03

**Authors:** Hakan Kaleli, Selman Demirtaş, Veli Uysal, Ioannis Karnis, Minas M. Stylianakis, Spiros H. Anastasiadis, Dae-Eun Kim

**Affiliations:** 1 Faculty of Mechanical Engineering, Automotive Division, Yildiz Technical University, Besiktas, Yildiz, 34349 Istanbul, Turkey; selmand@yildiz.edu.tr (S.D.); v_uysal@hotmail.com (V.U.); 2 Institute of Electronic Structure and Laser, Foundation for Research and Technology Hellas (FORTH), GR-70013 Heraklion, Crete, Greece; gkarnis@iesl.forth.gr (I.K.); spiros@iesl.forth.gr (S.H.A.); 3 Center for Nano-Wear, Department of Mechanical Engineering, Yonsei University, Seoul 03722, Korea; kimde@yonsei.ac.kr

**Keywords:** reduced graphene oxide, dispersion, engine oil additives, 5W-40, friction, friction coefficient, wear, tribological properties

## Abstract

We investigated the tribological behavior of commercialized, fully synthetic engine oil upon the incorporation of reduced graphene oxide in seven different concentrations between 0.01 and 0.2 wt %. Stability of the prepared samples was assessed by turbidimetry and dynamic light scattering measurements, and their tribological properties through a reciprocating tribometer, using a steel ball on special cut steel blocks. The addition of 0.02 wt % of reduced graphene oxide led to an improvement of the tribological behavior compared to the pristine engine oil, by significantly lowering the friction coefficient by 5% in the boundary lubrication regime. Both the surfaces and the reduced graphene oxide additive were thoroughly characterized by microscopic and optical spectroscopy techniques. We also verified that a protective layer was formed between the worn surfaces, due to the presence of reduced graphene oxide. Carbon accumulation and various additive elements such as Ca, Zn, S and P were detected on the rubbing surfaces of both the ball and the block through energy-dispersive X-ray spectroscopy. Finally, it was shown that the wear scar diameter on the surface of the steel ball was lower by 3%, upon testing the engine oil sample containing reduced graphene oxide at concentration 0.02 wt %, compared to the control sample.

## 1. Introduction

One of the most effective ways to tune friction is the use of a liquid lubricant. Liquid lubricants almost eliminate friction either by preventing sliding contact surfaces from severe or more frequent metal-to-metal contacts or by forming a low-shear, high-durability boundary film on the rubbing surfaces [1,2]. For example, depending on the sliding speed and other operating conditions, engine oils can effectively separate contact surfaces of rings and liners by reducing the frequency of direct metal-to-metal contact and, thus, reducing friction and wear. In case of metal-to-metal contact, any additive in these oils may form a low-shear, highly protective boundary film providing additional safety [1,2]. In short, low friction means fuel savings, while reduced wear implies longer durability [2,3]. Due to these solid and liquid lubricants, the components of today’s engines and other mechanical systems can be safely and smoothly operated for long times. In this context, graphene and its derivatives can serve as ideal solid or colloidal liquid lubricant additives due to their well-established lubricity, thermal stability, extraordinary electrical and mechanical properties and their impermeability to humidity and oxygen [4,5,6,7].

The capability of graphene-based materials to provide surface protection by reinforcing the properties of conventional lubricants, against friction and abrasive wear, when incorporated as additives, is mainly derived from two factors. On one hand, the existence of weak van der Waals forces in synergy with temperature dependent transitional and rotational motion between the flakes facilitate sliding between each other, thus, reducing shear resistance [5,7,8,9]. On the other hand, graphene-based materials passivate the rubbing surfaces, assisted by other additives of the lube oil [10,11], by forming continuous protective layers on the sliding surfaces, in case of uniform degradation (smooth surfaces), or by the growth of island shape coatings, when degradation is coarse [12]. Both factors contribute in a significant reduction of the friction coefficient (COF), while the unique mechanical strength of graphene derivatives can greatly shield surfaces against abrasion and corrosion [10,11]. This capability is extensively reported by recent tribological studies, revealing that graphene is able to lubricate in macroscale steel-on-steel sliding contacts very effectively under boundary lubrication conditions [4,13,14].

Although the incorporation of nanoadditives in lube oils is considered as a significant step towards the efficiency improvement of lubricants, a random addition of nanomaterials in engine oils may cause aggregation of the flakes; such agglomerates may even precipitate due to gravity [6,15]. Aggregation takes place whenever the Van der Waals forces between the nanomaterials are stronger than the repulsive ones [16] and the Brownian motion cannot overcome the attraction. When agglomeration occurs in the nanomaterials’ dispersions, sedimentation is sped up, while friction performance is negatively affected [17,18]. Hence, long-term stability still remains challenging towards the formulation of highly efficient and reliable nanolubricants in order not to settle down after a long time of storage, ensuring negligible mechanical failure (wear) between worn surfaces [16,19].

Indeed, the most relevant studies report on a critical concentration of graphene-based additives in lube oils ranging from 0.01% to 1%, which sustains a tradeoff between COF improvement and long-term stability [10,20,21,22,23]; the optimum concentration varies depending on the nature of the graphene based additive, such as variation of functional groups, number of layers, flake size and the type of the sliding contacts [24]. An extensive investigation on the relative factors to improve the stability of lube oils incorporating nanoparticles is imperative in order to produce highly efficient lubricants that can reduce friction and mechanical failure between worn surfaces, which are considered as the main causes of energy dissipation in automobile engines.

To this end, we demonstrate for the first time the improvement of the tribological behavior of a conventional fully synthetic engine oil 5W-40, by Shell, through the addition of rGO of different contents, in a range from 0.2 to 0.01 wt %, assisted by ultrasonication. rGO is an atomically thin sheet of carbon atoms, processed by the reduction of graphene oxide (GO), through various methods in order to reduce the oxygen functional groups [25,26,27,28,29], and is widely investigated for various emerging applications [30,31,32,33]. Here, we investigate the lubrication mechanism, which finally is due to the formation of a protecting layer on the sliding surfaces; carbon accumulation was detected on the surfaces, which originated from the rGO, and various elements such as Ca, Zn, S and P, because of other additives in the Shell 5W-40 engine oil. The prepared “hybrid” nanolubricant 5W-40 containing 0.02 wt % rGO, remained stable for more than 6 months, while it resulted in lower COF compared to the reference 5W-40 engine oil by 5%, as estimated with the use of a tribometer. It should be noted that friction tests were conducted according to the active standard test method (ASTM) G181 [34], attesting antifriction and antiwear properties of rGO when embedded in lubricants.

## 2. Materials and Methods

### 2.1. Reagents and Consumables

Graphite powder, sodium nitrate, potassium permanganate, hydrogen peroxide 30%, hydroiodic acid (HI) 55%, sodium bicarbonate and acetone were purchased from Sigma-Aldrich. Sulfuric acid 95–98% and glacial acetic acid were purchased from Honeywell Advanced Materials (NJ, USA) and Merck (Darmstadt, Germany), respectively. 5W-40 synthetic multigrade engine oil, provided by Shell Co. (Istanbul, Turkey) (Table 1), is fully synthetic, enriched by very low contents (confidential) of some chemical elements such as Ca, Zn, P, S and Mg. Its specifications conform to API SN/CF; ACEA A3/B3, A3/B4; BMW LL-01; MB 229.5, 226.5; VW 502.00/505.00; Porsche A40; Renault RN0700, RN0710; PSA B71 2296, Ferrari; Fiat 9.55535-Z2 and Chrysler MS-10725. High-carbon, high-chromium tool steel specimens alloyed with molybdenum and vanadium were supplied by Uddeholm (Oldbury, West Midlands, UK) (Sverker 21-AISI D2, dimensions: 13 mm × 10 mm × 10 mm, hardness: 210 HB Ball, 100CrMn6 steel ball, d = 8 mm, hardness: 60-66 HRC).

### 2.2. Materials’ Preparation Procedure

#### 2.2.1. Preparation of rGO

According to a typical procedure, GO (100 mg) synthesized by a modified Hummers method [27] was reduced by a mixture of glacial acetic acid and hydroiodic acid (AcOH/HI), as reported elsewhere [27,31]. This method yields rGO in a powder form.

#### 2.2.2. Preparation of the Engine Oil (5W-40) Samples Incorporating rGO as an Additive

**Figure 1:**
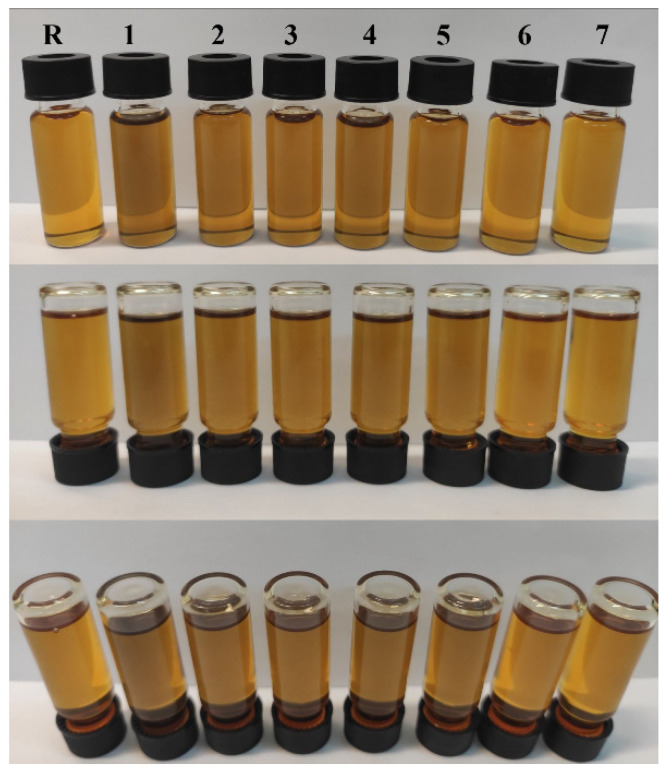
Photographs of the reference 5W-40 synthetic oil (R) and the prepared oil samples incorporating rGO at different concentrations (R—0 wt %, 1—0.2 wt %, 2—0.1 wt %, 3—0.08 wt %, 4—0.06 wt %, 5—0.04 wt %, 6—0.02 wt % and 7—0.01 wt %), photographed from different angles.

In a typical procedure, engine oil (20 mL/10 g) was added in a clean vial (22 mL). Then, rGO was incorporated in different mass ratios (0.2–0.01%) and the mixture was homogenized for 30 min, utilizing an ultrasonic probe assisted process. Table 2 summarizes the preparation parameters of the engine oil samples containing different concentrations of rGO. All samples were centrifuged (Sigma 2-16P) at 4500 rpm (1856× *g*) for 30 min, in order to remove any aggregate/sediment and to increase their long-term stability (Figure 1), albeit no sedimentation was occurred thereupon the centrifugation.

### 2.3. Tribological Characterization

**Figure 2:**
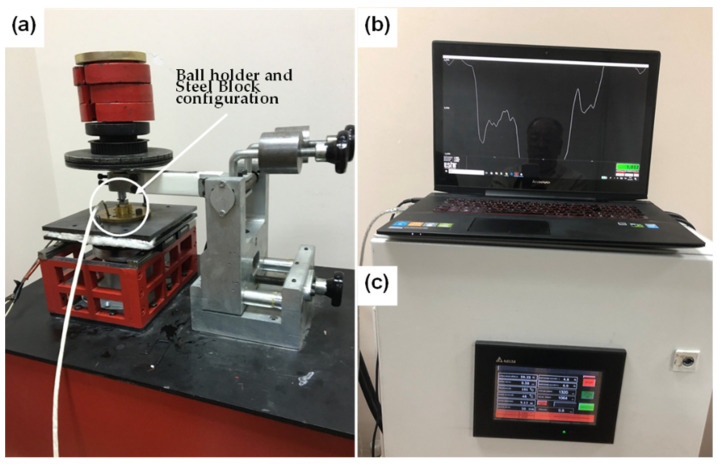
Reciprocating tribotest setup, (**a**) a general view, (**b**) recording of accurate friction force data measurement and (**c**) heating and temperature control and monitoring.

**Figure 3:**
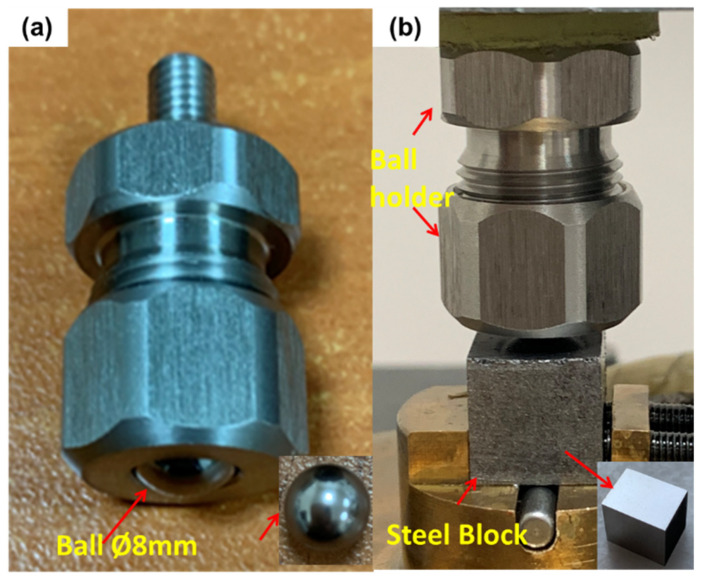
Ball and block specimen configuration: (**a**) ball placed in the holder and (**b**) ball holder and steel block integrated configuration.

Tribological performance of the reference Shell 5W-40 engine oil was assessed according to formal, well-established test parameters since 2000, and compared with the prepared dispersions (0.2–0.01 wt % rGO content), as listed in Table 2, utilizing a customized reciprocating tribometer setup, as depicted in Figure 2. The tribometer is driven by a servomotor, and 800 data (values) per second were collected from the load cell, using a data logger, directly connected to a monitor. Each stroke lasted 0.8 s (640 data per second) and in this way the average COF of the wear track during the measurement was estimated. All tests were carried out under boundary lubrication conditions at 100 °C. Normal load during the tribological measurements was set to be 60.5 N. Sliding speed or reciprocating velocity and stroke were 0.055 m/s and 8 mm, respectively. The temperature of the block was set up to 100 °C, using an advanced heater, equipped with a digital controller mounted on the setup. To run a 21 min tribotest measurement, three drops from each sample were dropped on the surface of the steel block. Then, the 100CrMn6 steel ball rubbed on it under boundary lubrication conditions, as displayed in Figure 3. Data acquisition was done by a specific software, developed using MATLAB (R2020b, MathWorks, MA, USA), which was used in order to filter any vibrational noise and to calculate the average COF, by plotting the final COF curve, as a function of time.

### 2.4. Characterization Methods

The dispersion samples were prepared through an ultrasonic homogenizer by J.P. SELECTA S.A. (Barcelona, Spain) (model CY500 with ultrasonication energy of 20 Hz). Attenuated total reflection Fourier transform infrared (ATR FTIR) measurements (absorbance) were carried out with a Bruker (Billerica, MA, USA) Vertex 70v FTIR vacuum spectrometer equipped with a A225/Q Platinum ATR unit with single reflection diamond crystal, which allows the infrared analysis of unevenly shaped solid samples and liquids through total reflection measurements, over the spectral range of 4000–700 cm^−1^. Raman measurements were performed at room temperature (RT) using a Horiba (Kyoto, Japan) LabRAM HR Evolution confocal microspectrometer, in backscattering geometry (180°), equipped with an air-cooled solid-state laser operating at 532 nm with 100 mW output power. The laser beam was focused on the samples using a 10× Olympus microscope objective (numerical aperture of 0.25), providing 55 mW of power on each sample. Average particle diameter and zeta potential measurements of the rGO dispersions were performed utilizing a Malvern (Malvern, UK) Nano ZS (Nano ZS90) instrument. XRD patterns were collected on a Bruker (Billerica, MA, USA) D8 Advance X-ray diffractometer, using Cu Kα radiation (λ = 1.5406 Å). Morphology characterization was performed through a fully digital optical microscope, using a Nanobender 3D HR optical profilometer, with resolution 6400/rotation equipped with a -P-CAMn camera (2 MP and 5 MP). SEM images were taken through a JEOL JSM-7000F (Tokyo, Japan) field emission scanning electron microscope, while TEM images were extracted through a JEOL JSM2010 (Tokyo, Japan) transmission electron microscope operated with accelerating voltage at 200 kV. Turbidimetry measurements were taken using a HI93703 Microprocessor Hanna Instruments (Woonsocket, RI, USA) Turbidimeter. Tribological properties of the samples were evaluated using the customized reciprocating tribometer discussed above.

## 3. Results and Discussions

### 3.1. rGO Analysis

rGO was thoroughly characterized by several techniques before its incorporation as additive in the pristine 5W-40 Shell engine oil. In Figure S1a,b, typical ATR FT-IR absorbance and Raman spectra of rGO are shown. In the ATR FT-IR spectrum, few weak absorption bands are displayed, proving the successful synthesis of rGO upon the HI-assisted reduction of its precursor GO [27]. The Raman spectrum follows the typical trend of rGO, presenting the D peak at 1343 cm^−1^, the G peak at 1573 cm^−1^ and the 2D peak at 2684 cm^−1^ [35]. Moreover, the XRD pattern of rGO displays a characteristic broad peak at 24.7° and a smaller one at 42.7°, corresponding to an interlayer distance of 3.5 A [36], as depicted in Figure S1c. To further investigate the morphology of the rGO wrinkled structured flakes, SEM and TEM microscopy were carried out as demonstrated in Figure S2a,b, respectively.

### 3.2. Evaluation of 5W-40 Oil Samples Incorporating rGO

#### 3.2.1. Turbidimetry Measurements

**Figure 4:**
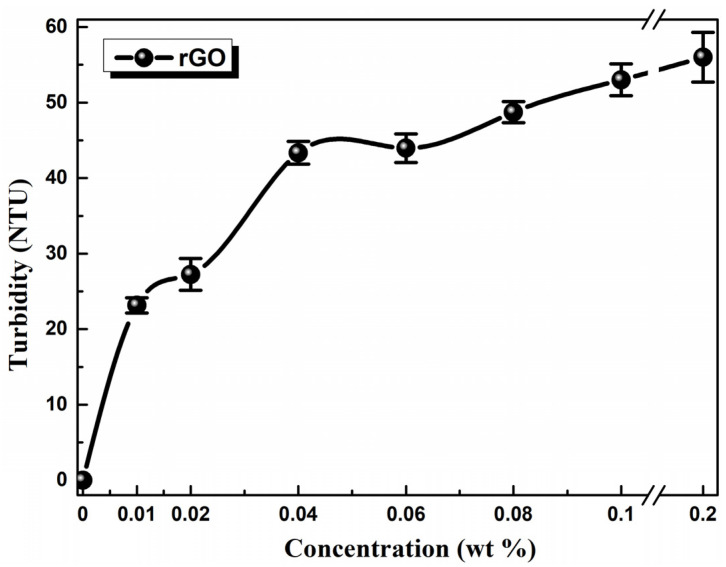
Turbidimetry measurements of the reference 5W-40 engine oil and the prepared dispersions containing rGO as additive from 0.2% to 0.01 wt %. The line is to guide the eye.

Figure 4 demonstrates the turbidity of the oil samples as a function of rGO concentration. It is obvious that turbidity increased rapidly for oil samples of low rGO concentrations, whereas it shows a weaker increase for high concentrations. This fact implies the presence of larger size particles due to aggregation of rGO flakes [37]. Consequently, mild sedimentation was observed for samples rGO5–rGO1 after 3–4 months, while the rGO7–rGO6 ones presented longer-term stability (>6 months).

#### 3.2.2. Size and Zeta Potential Measurements

The size of the rGO flakes and their zeta potential in suspension were estimated with dynamic light scattering (DLS) measurements at a 90° scattering angle at 25 °C. Only measurements for sample rGO7 were meaningful, since the concentration of the other samples was too high. As estimated, the average diameter of the rGO flakes was 531 nm, with a PDI of 0.61, while the zeta potential was −30 mV, implying high stability of the rGO7 sample [27,38].

### 3.3. Surface Analysis

**Figure 5:**
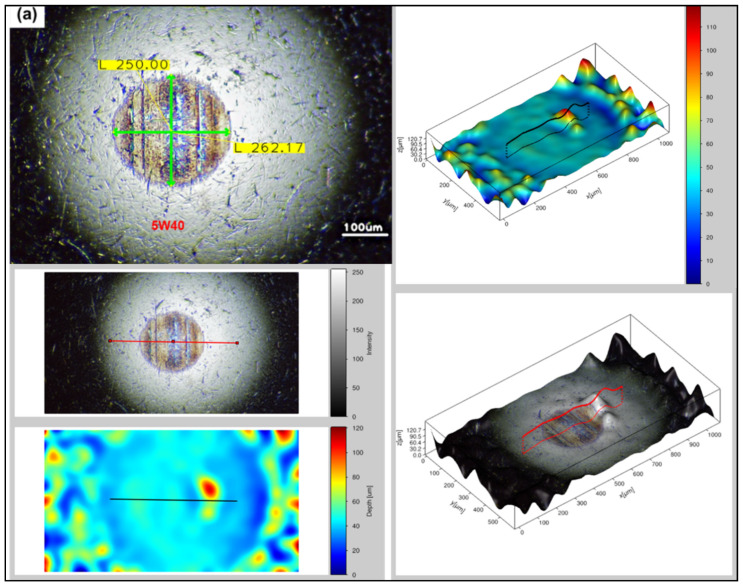

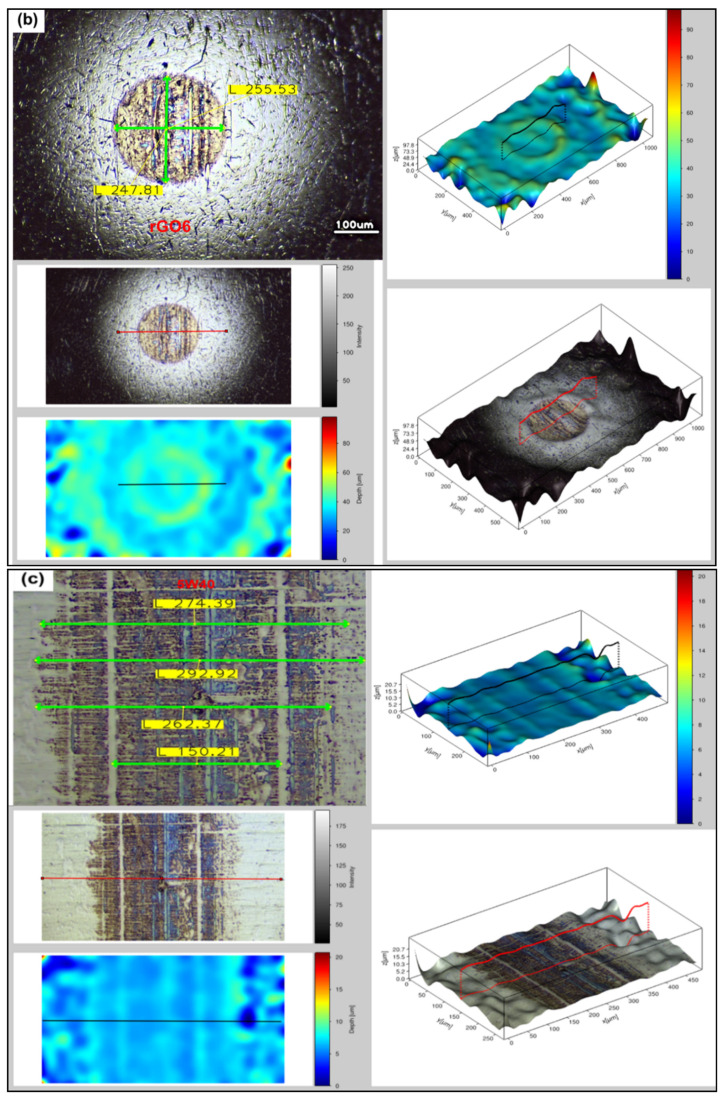

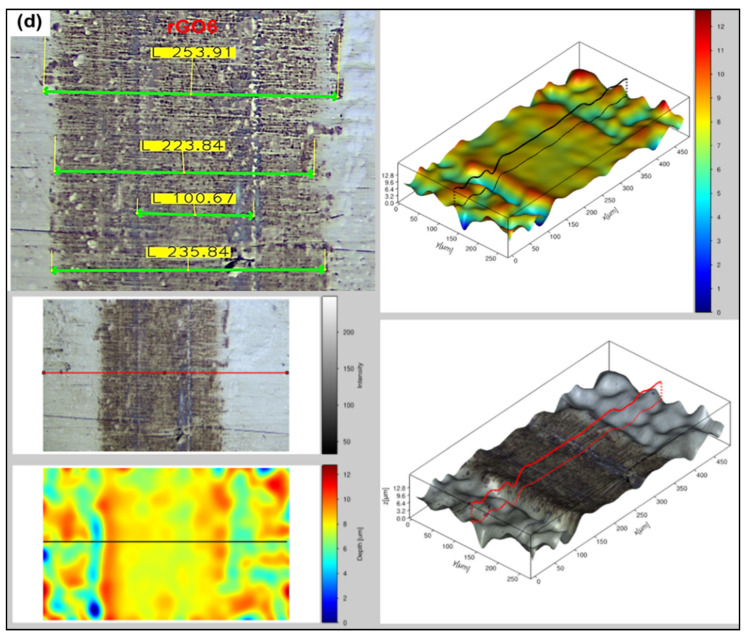
Optical microscopy images of the rubbing balls (**a**,**b**) and blocks (**c**,**d**) taken after the first measurement, to investigate the wear scar and roughness after testing (**a**,**c**) 5W-40 engine oil and (**b**,**d**) rGO6 sample.

Surface analysis of the steel blocks sliding pairs (see Figure 3) has been carried out through a fully digital optical microscope, as well, in order to compare the degree of deformation occurred upon testing the reference and the rGO6 samples, while 2D/3D roughness parameters were measured by a 3D high resolution optical profilometer. According to the first measurement performed, as shown in Figure 5, the wear scar on the ball and block, after testing the sample rGO6 (b,d), is totally smaller than the respective one, corresponding to the reference (a,c), exhibiting a reduction by 3% in the wear scar diameter of the ball. More specifically, the wear scar diameter on the ball was reduced from 262.17 to 255.53 µm horizontally and from 250 to 247.81 µm vertically, while the width of the wear track of the block decreased from 292.92 to 253.91 μm as well. In addition, the surfaces of the steel sliding pairs upon testing rGO6 exhibited an average roughness (R_a_) of 3.256 μm for the ball and 0.648 μm for the steel block, compared to the rougher ones corresponding to the reference, which displayed an R_a_ of 6.129 μm and 0.762 μm, respectively. The respective raw data are also demonstrated in Figure S3. It should be also noted that the wear scar on the ball and block corresponding to the second and third measurements followed the same trend as the first one, as presented in Figure S4a,b, respectively. In particular, the wear scar diameter on the ball was decreased from 269.92 to 259.96 µm horizontally and from 258.85 to 247.79 µm vertically, while the width of the wear track of the block dropped off from 243.35 to 236.73 μm in case of the second measurement. According to the third measurement, the wear scar diameter on the ball lessened from 276.90 to 266.88 µm horizontally and 264.38 to 250.36 µm vertically, while the width of the wear track of the block also declined from 268.81 to 232.23 μm. It should be noted that in order to compare the wear track on the surface of the balls, corresponding to the reference and rGO6 samples tests, we compared their respective vertical and horizontal diameters (light-green secant lines). On the other hand, to compare the wear track on the surface of the blocks, we selected similar regions of the wear scars of the two blocks to draw four light-green straight lines at different positions of the rubbed area of the blocks. In that way, we compared the highest width value of each block.

**Figure 6:**
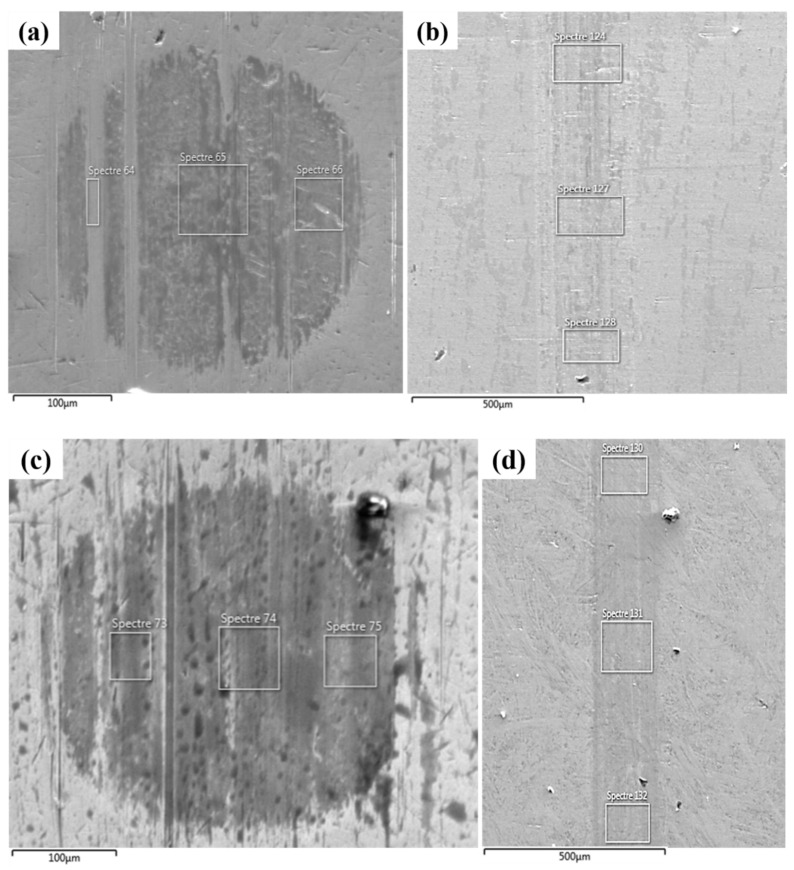
SEM images depicting the wear scar corresponding to: (**a**) the ball and (**b**) the block tested with the reference 5W-40 engine oil and (**c**) the ball and (**d**) the block tested with the rGO6 sample. The white square frameworks correspond to the regions of the samples where EDS analysis was conducted. EDS spectra are provided as Supplementary Materials in Figure S5.

SEM images (Figure 6) supported by energy-dispersive X-ray spectroscopy (EDS) (Figure S5) of the reference and the rGO6 samples were undertaken to investigate the degree of deformation of the rubbing surfaces, and the extended presence of carbon originating from rGO through EDS mapping. It is apparent that surface deformation for both the ball and the block parts after testing the rGO6 sample was significantly lower compared to the 5W-40 engine oil. On one hand, this fact is attributed to self-lubricating behavior of rGO [39]. On the other hand, according to the elemental analysis, the carbon content on rubbing surfaces was almost double, while the Fe content was reduced for rGO6 sample (Figure S5c,d) as compared to the 5W-40 sample (Figure S5a,b), as also listed in Table 3. This result confirms the existence of a protective coating on the rubbing surfaces, due to the rGO additive, which provides a more effective lubrication between the sliding parts. Therefore, it is apparent that rGO plays a key role towards the diminution of friction and the wear track reduction, contributing to surface protection against shear stress through the delamination effect [20].

### 3.4. Tribological Characterization

The tribological properties of the samples were evaluated using a steel ball (100CrMn6 or 52100) on special cut steel blocks (Sverkel 21-AISI D2), as previously described. The tribological tests were carried out in triplicate, obtaining very similar results in all cases, confirming that the wear scar on the ball and block, after testing the sample rGO6 was smaller than in the case of testing the reference engine oil.

**Figure 7:**
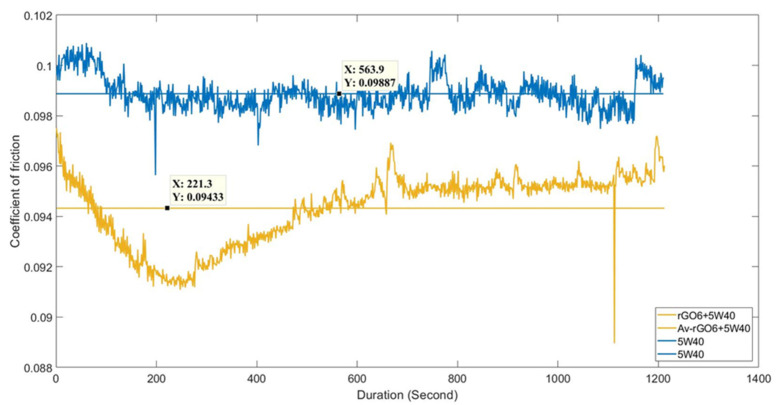
Friction coefficient (COF) values obtained after the first experimental reciprocating tribotest of the reference 5W-40 (blue, top) and rGO6 sample (brown, bottom), as a function of testing time.

Figure 7 shows the variation of COF values as recorded during the first experimental reciprocating tribotest of the reference 5W-40 and the rGO6 samples. The second and the third one are included in the Supplementary Materials section, as Figure S6a,b, respectively. The horizontal lines represent the average COF value, which is automatically plotted by the MATLAB assisted specific software of the reciprocating tribotest setup. The average COF value exhibited a reduction by almost 5%, as estimated upon the completion of the experimental reciprocating tribotests in triplicate.

**Figure 8:**
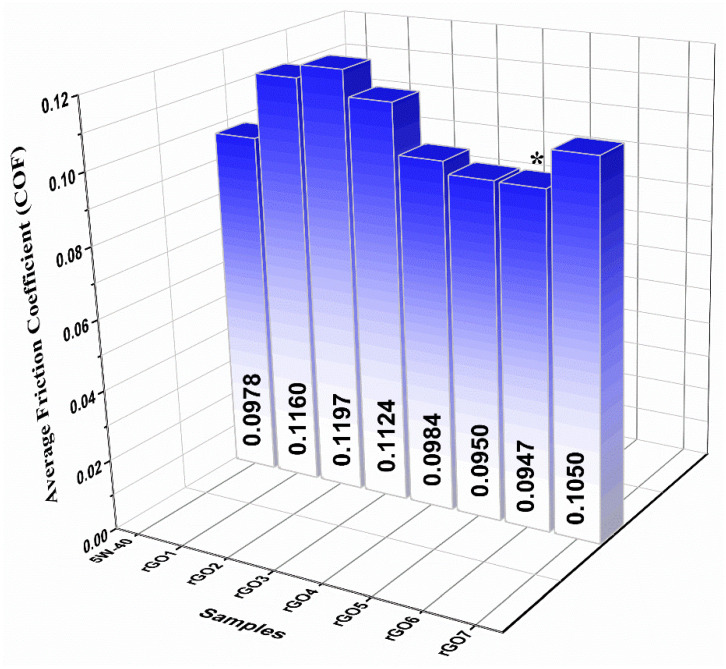
Average COF values histogram of the reference 5W-40 engine oil and the samples incorporating reduced graphene oxide (rGO) 0.2–0.01 wt %. The sample exhibited the lowest average COF value is denoted by an asterisk. The standard deviation in these data are 1%.

The histogram in Figure 8 represents the extracted average COF values of the samples, including the reference 5W-40 engine oil. Among the samples, sample rGO6 (0.02 wt %) presented superior tribological properties by exhibiting the lowest COF value. It is evident that additional protection to rubbing surfaces from wear was provided, due to the formation of a protective layer, represented by colorful regions, as displayed below in the optical microscopy images (Figure 5) [40]. The sample rGO7 exhibited higher COF than rGO4, rGO5 and rGO6, although its rGO concentration was lower. This is possibly due to the fact that rGO content was not enough to form a uniform protective coating between the sliding parts.

## 4. Conclusions

Tribological properties of commercialized, fully synthetic engine oil (Shell 5W-40) were thoroughly assessed upon the addition of rGO for seven different concentrations of rGO ranging from 0.01% to 0.2 wt %. Stability of the prepared samples was assessed, by turbidimetry and zeta potential measurements. We concluded that protective coatings were formed onto the worn surfaces upon using the rGO6 sample (0.02 wt % rGO content), contributing to a good surface passivation, since COF decreased by 5%, while the wear scar diameter of the ball was reduced by 3%, as compared to the control sample. To conclude, our study highlights the potential of HI assisted rGO as a lubricant additive, towards sufficient protection of rubbing surfaces against degradation through wear and friction elimination, leading to fuel energy saving [41]. These very promising results make us keen on conducting controlled trial runs of rGO6 sample in real engines within the next months.

## Figures and Tables

**Table 1:** The properties of 5W-40 synthetic engine oil.

Properties	
Total Base Number (TBN)	11.28 mg KOH/g
Flash point	217 °C
Viscosity @100 °C	13.59 cSt
Viscosity @40 °C	84.27 cSt

**Table 2:** Samples classification

Sample	Oil (ml)	Oil (g)	rGO (mg)	rGO (wt %)
**R**	20	10	0	0
**1**	20	10	20	0.2
**2**	20	10	10	0.1
**3**	20	10	8	0.08
**4**	20	10	6	0.06
**5**	20	10	4	0.04
**6**	20	10	2	0.02
**7**	20	10	1	0.01

**Table 3:** An overview of the elemental analysis of the wear scar of the rubbing parts by EDS.

Spectrum No	Sample Name	C	O	Al	Si	P	Ca	Mn	Fe	Zn	Total
64	5W-40 ball	5.49	0.86	0	0.25	0.1	0.01	0.49	92.44	0.36	100
65		6.06	4.69	0.09	0.21	0.72	1.36	0.51	84.03	2.33	100
66		5.94	2.95	0.04	0.25	0.46	0.81	0.59	87.66	1.29	100
73	rGO6 ball	8.94	7.54	0.07	0.18	1.28	1.81	0.39	76.02	3.78	100
74		11.16	5.52	0.13	0.28	0.43	1.65	0.54	79.16	1.13	100
75		7.61	4.73	0.02	0.23	0.42	1.53	0.42	83.66	1.38	100
124	5W-40 block	6.44	3.76	0.08	0.34	0.55	0.92	1.34	83.47	3.1	100
127		7.69	3.67	0.07	0.37	0.42	0.86	1.82	82.01	3.09	100
128		7.72	3.75	0.08	0.36	0.51	0.82	2.04	81.69	3.02	100
130	rGO6 block	9.85	3.73	0.14	0.31	0.3	0.86	1.76	81.58	1.47	100
131		11	3.7	0.11	0.28	0.22	0.75	1.75	80.47	1.72	100
132		11.53	3.74	0.07	0.27	0.24	0.89	2.13	79.76	1.37	100

All element content is expressed as atomic percentage (%).

## Data Availability

Data is contained within the article or supplementary material.

## Supplementary Materials

The following are available online at https://www.mdpi.com/2079-4991/11/2/386/s1, Figure S1: (a) ATR FT-IR and (b) Raman spectra and (c) XRD pattern of rGO; Figure S2: SEM and TEM images of rGO; Figure S3: Roughness measurements raw data of the ball and block parts after testing the reference and rGO6 samples; Figure S4: Optical microscopy images of the wear scar on the steel balls and blocks taken after the (a) second and (b) third measurements, after testing the reference and rGO6 samples; Figure S5: EDS spectra of the ball and block parts after testing the reference and rGO6 samples; Figure S6: COF values obtained after the second and the third experimental reciprocating tribotests of the reference 5W-40 and rGO6 sample.
