# Supplementary material for: Tribological Performance Investigation of a Commercial Engine Oil Incorporating Reduced Graphene Oxide as Additive
Source: Nanomaterials (Basel). 2021 Feb 3;11(2):386. doi: 10.3390/nano11020386 (PMC7913578; doi:10.3390/nano11020386)
Supplement: Supplementary file 1 [file nanomaterials-11-00386-s001.pdf]

nanomaterials

Article

Tribological Performance Investigation of a Commercial Engine Oil Incorporating Reduced Graphene Oxide as Additive

Supporting Information

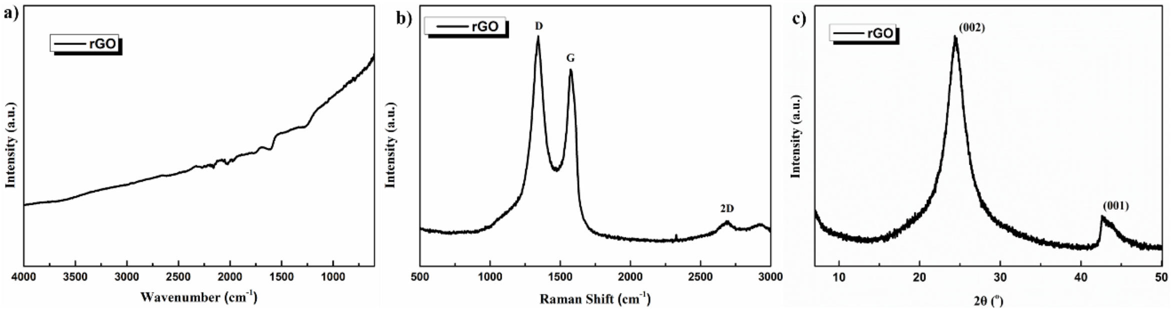

Figure S1. ATR FT-IR (a) and Raman (b) spectra and XRD pattern (c) of the rGO used in this study.

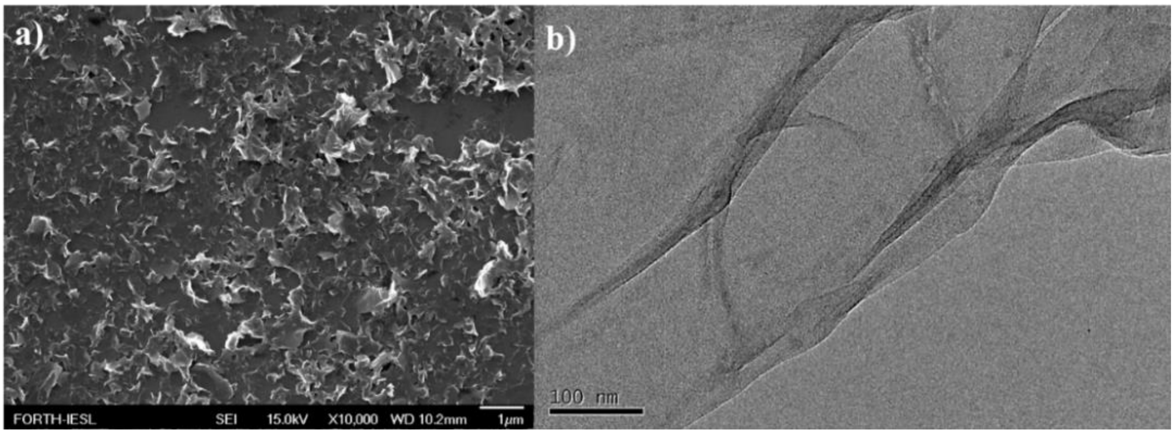

Figure S2. a) SEM and b) TEM images of the rGO used in this study.

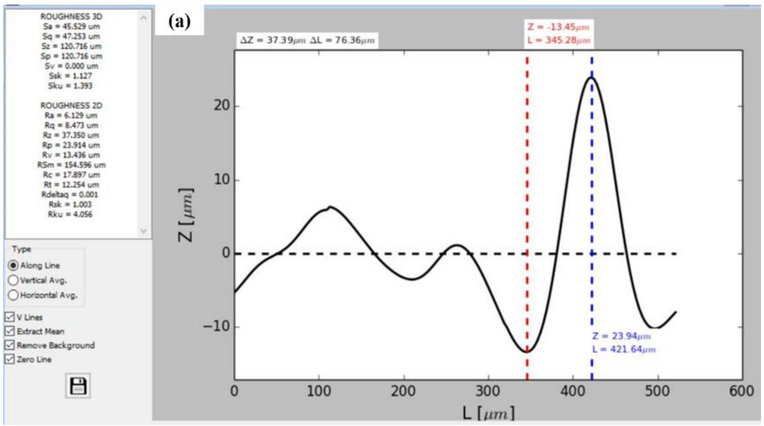

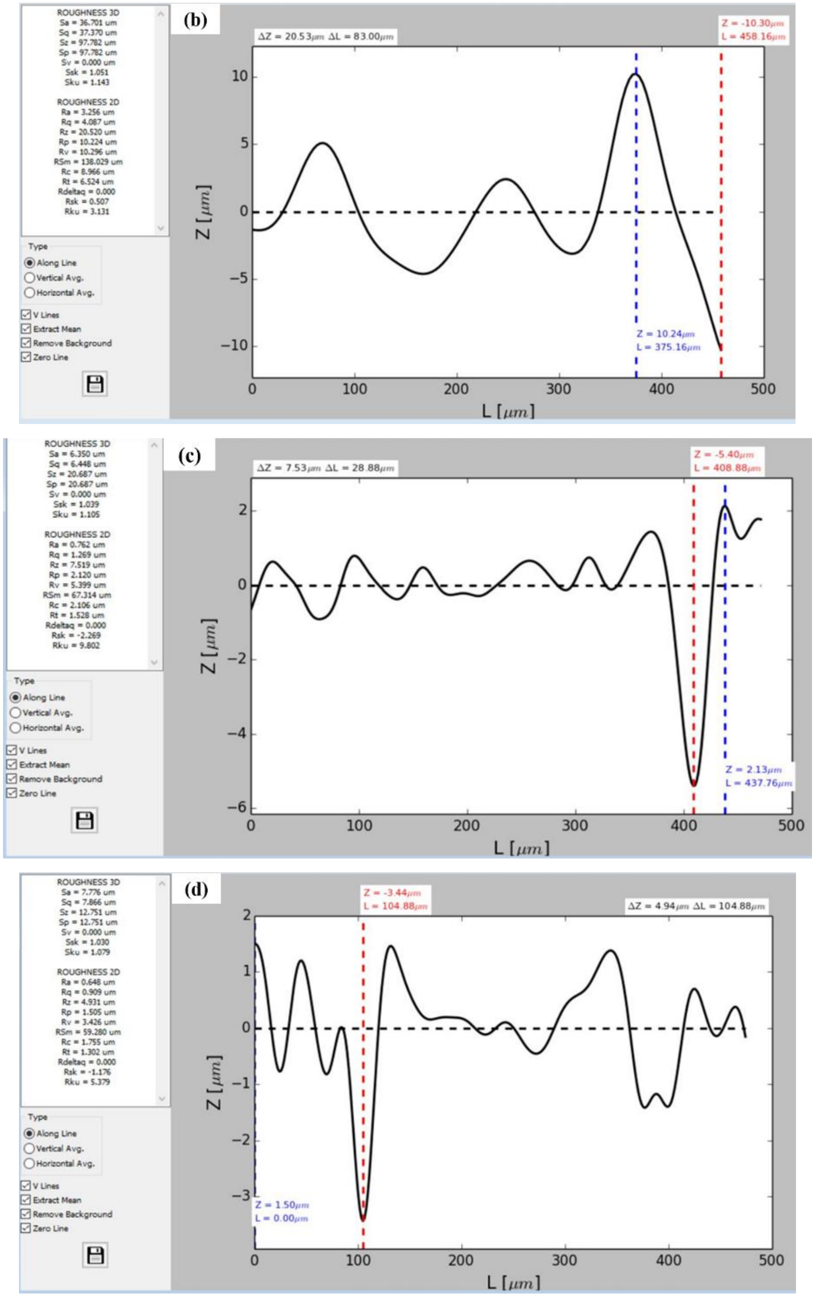

Figure S3. Roughness measurements raw data of the ball parts (a,b) and blocks (c,d) after testing with the 5W-40 engine oil (a,c) and with the rGO6 samples (b,d).

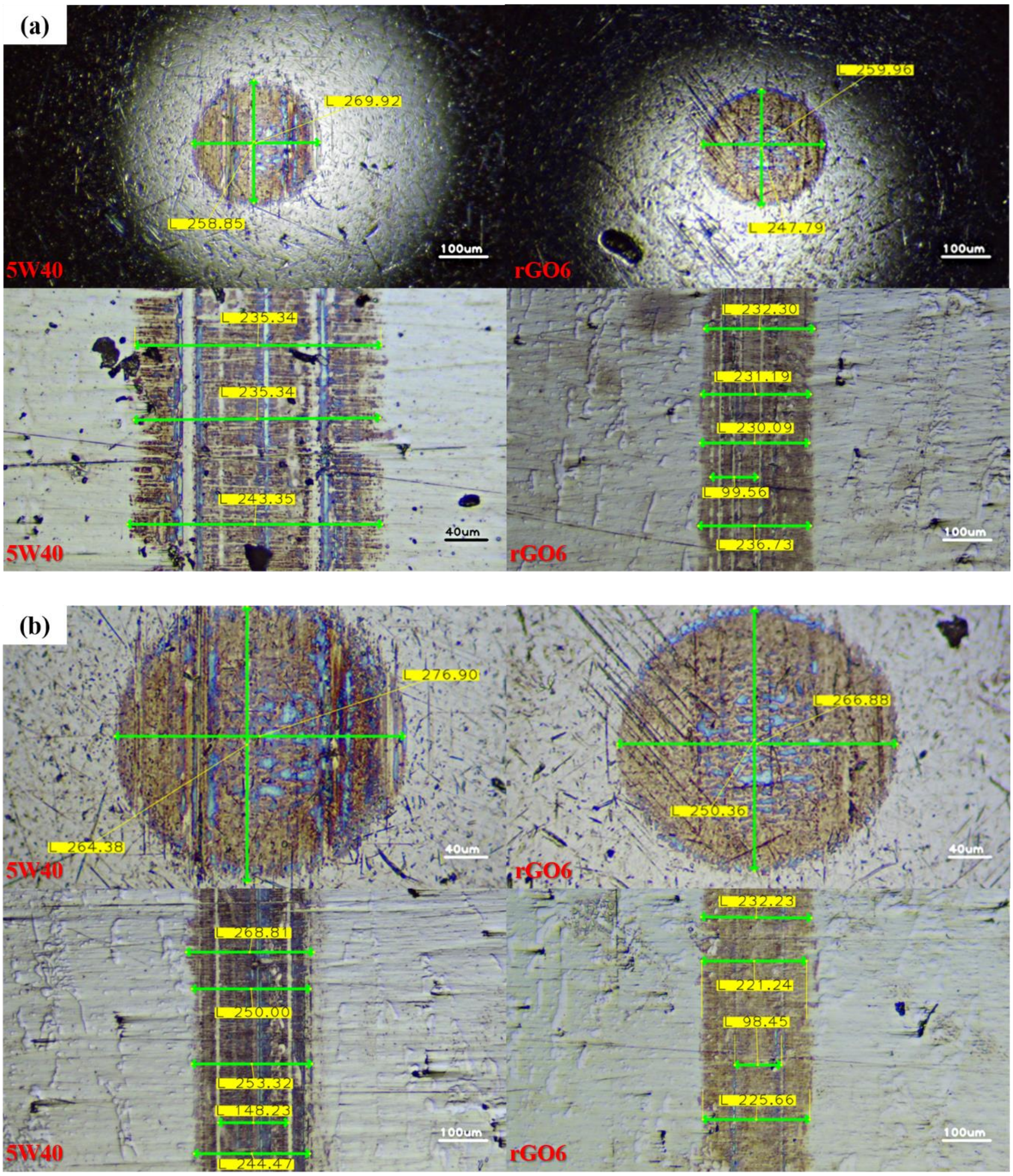

Figure S4. Optical microscopy images of the wear scar on the balls and steel blocks corresponding to a) the second and b) the third measurements, after testing the reference 5W-40 and rGO6 samples, respectively.

(a)

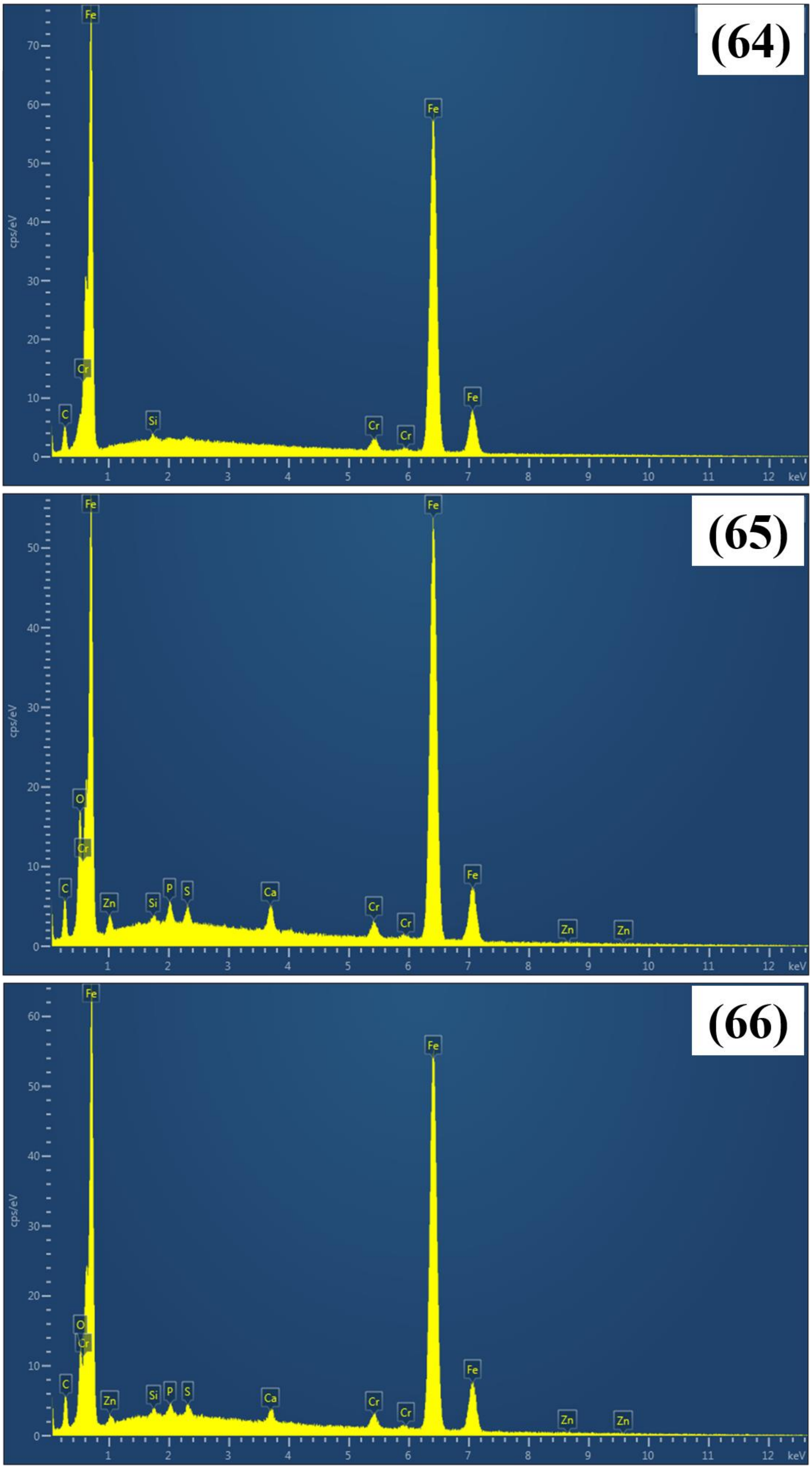

(b)

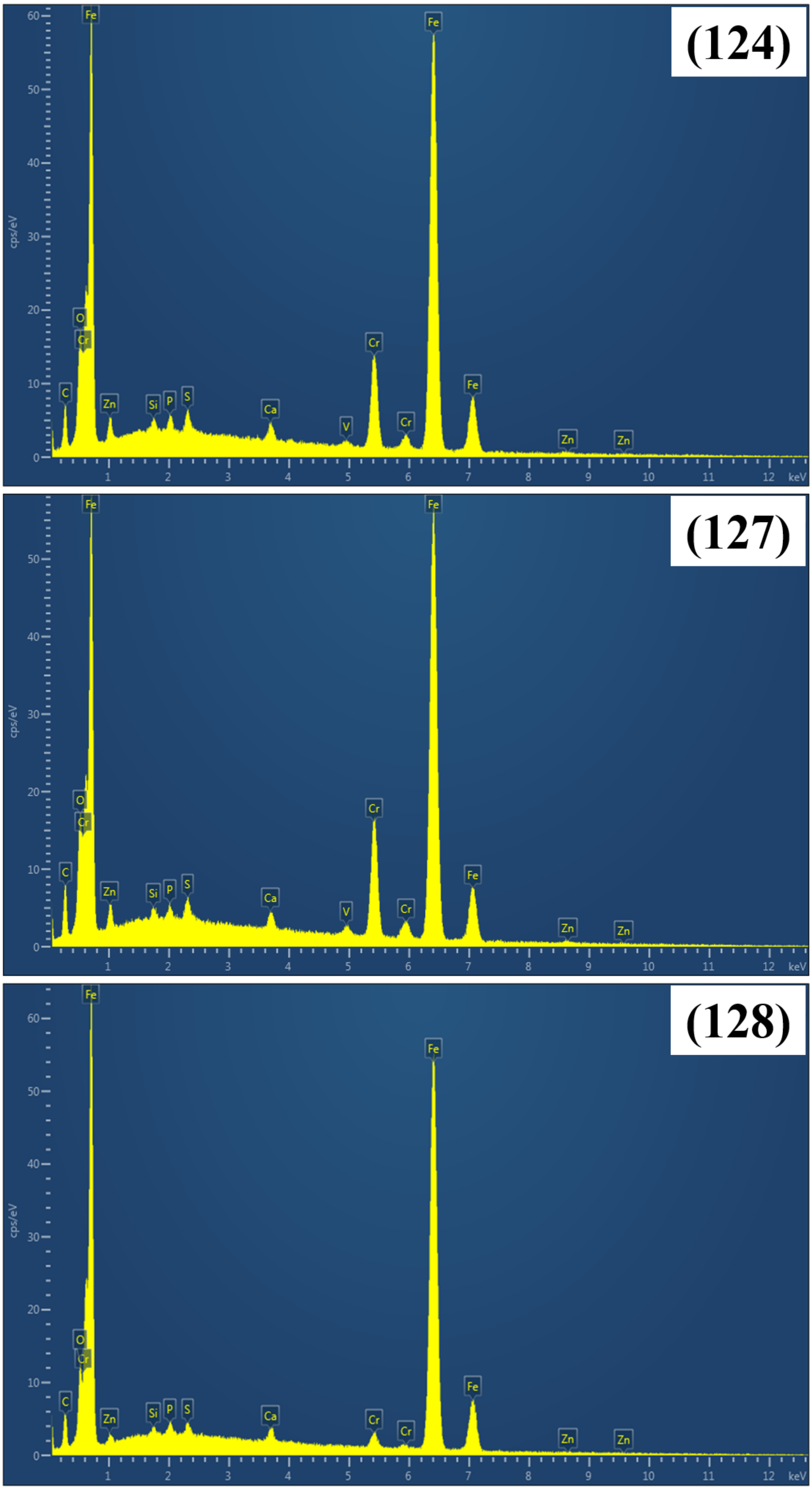

(c)

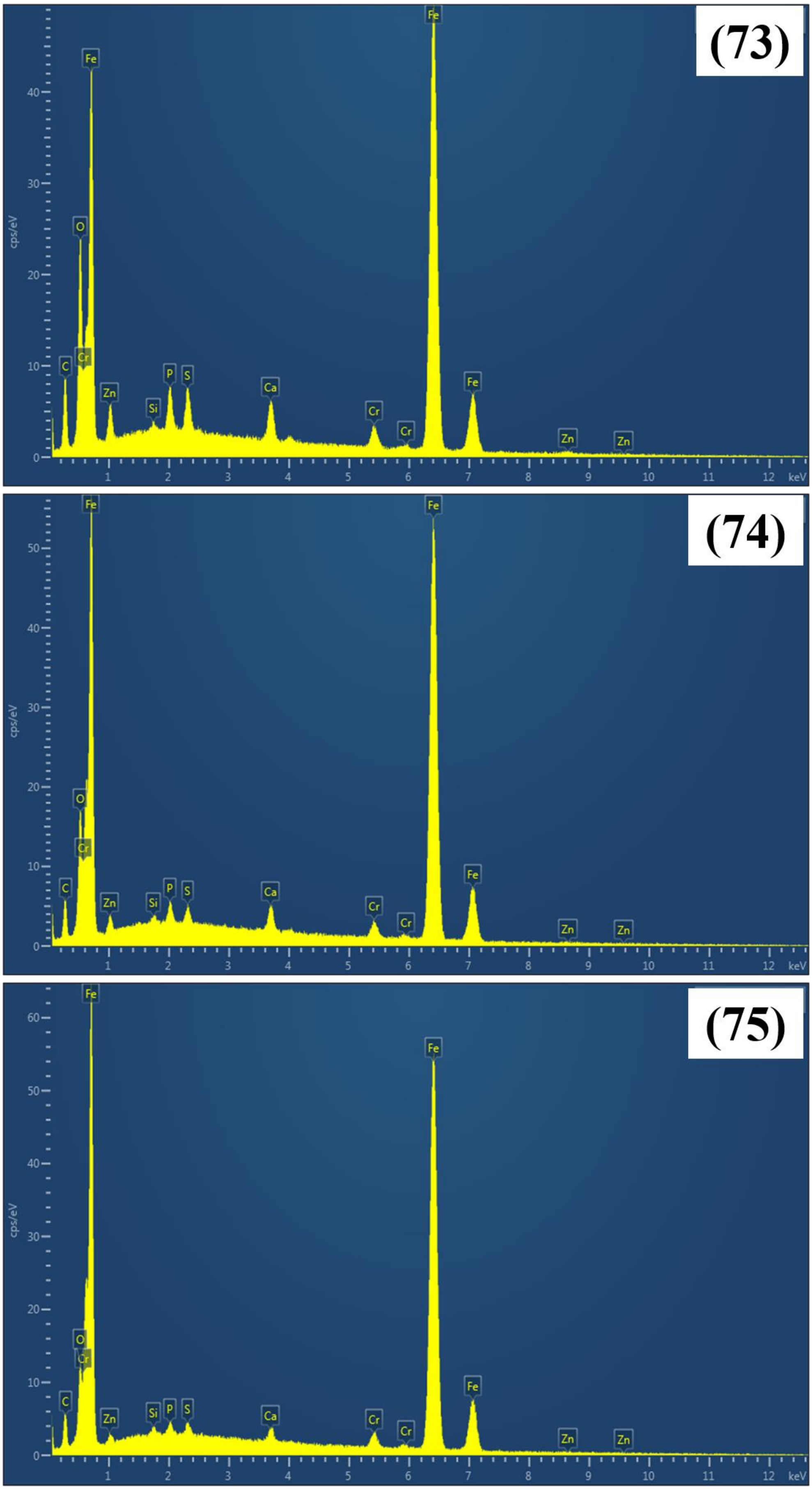

(d)

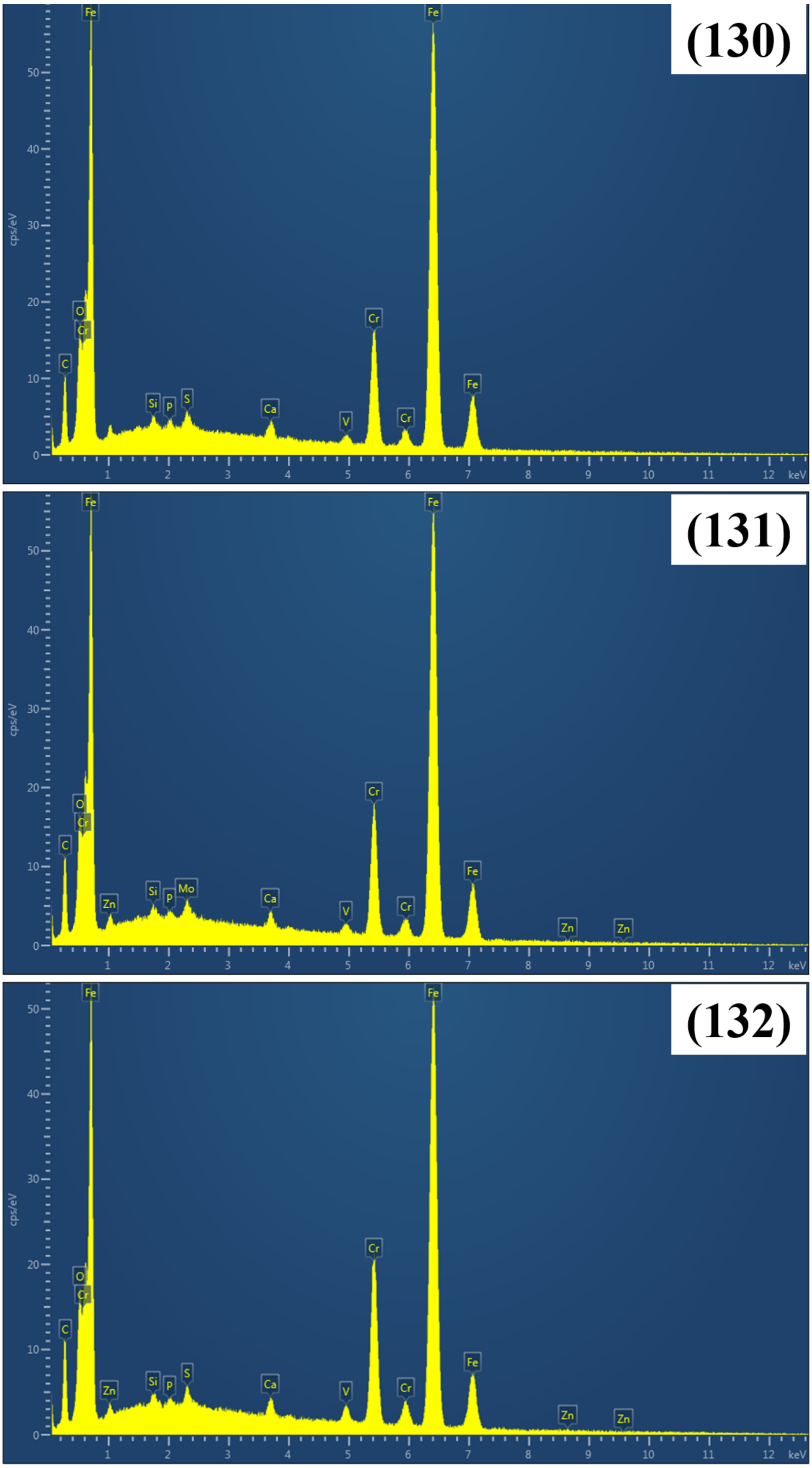

Figure S5. EDS spectra of (a) the ball and (b) the block tested with the reference 5W-40 engine oil and (c) the ball and (d) the block tested with the rGO6 sample. The numbers in the white frames correspond to the EDS spectrum No.

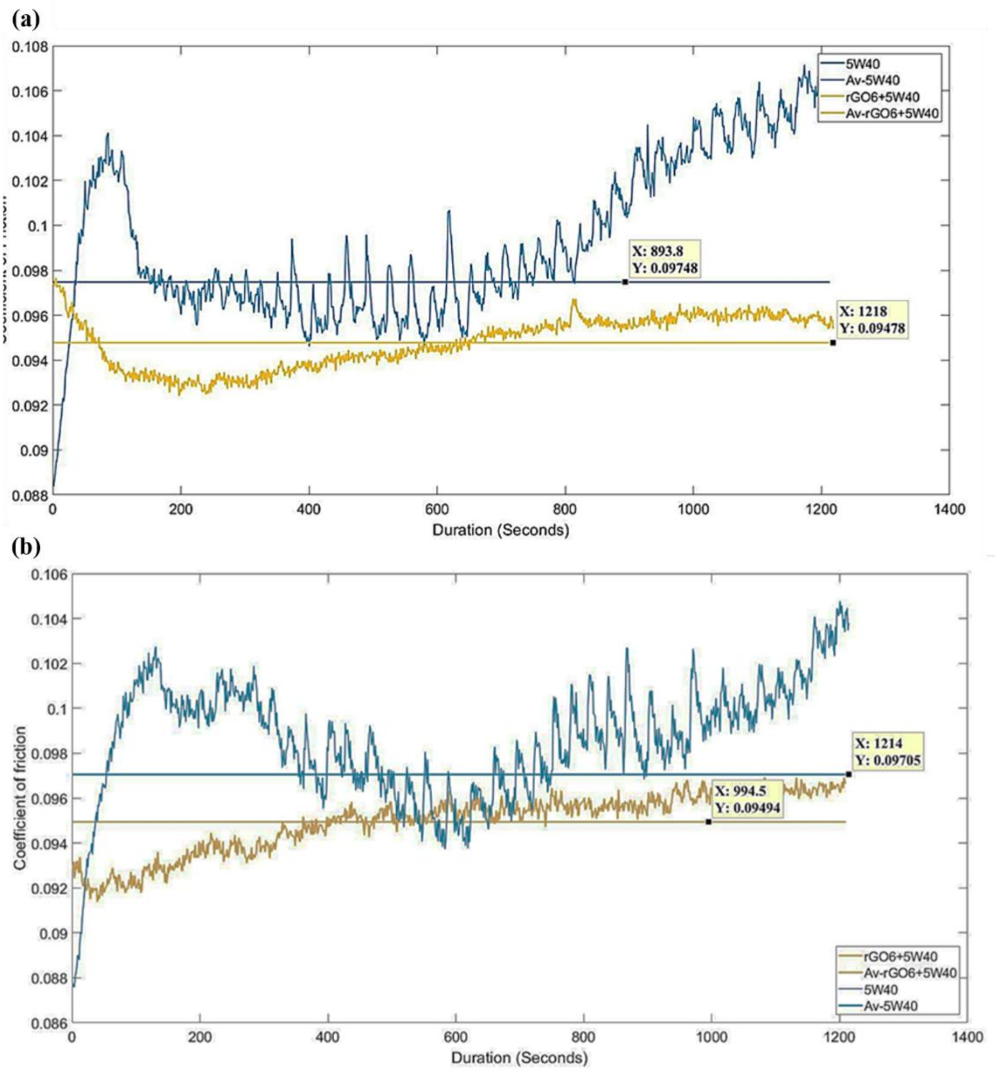

Figure S6. COF values obtained after the second (a), as well as the third (b) experimental reciprocating tribotests of the reference 5W-40 (blue, top) and rGO6 sample (brown, bottom), as a function of testing time.
